# Fever clinic construction and management targeted to prevention and control of healthcare-associated respiratory viral infections in Jiangsu, China: A cross-sectional observational study

**DOI:** 10.1371/journal.pone.0297133

**Published:** 2024-02-01

**Authors:** Yue Yang, Bo Liu, Ya-Jun Wen, Zhan-Jie Li, Yong-Xiang Zhang, Gen-Ming Zhao, Bi-Jie Hu, Wen-Sen Chen, Wei-Hong Zhang

**Affiliations:** 1 Shanghai Institute of Infectious Disease and Biosecurity, Fudan University, Shanghai, China; 2 Department of Infection Control, The First Affiliated Hospital of Nanjing Medical University, Nanjing, Jiangsu, China; 3 Department of Infectious Diseases & Hospital Infection Management, Zhongshan Hospital, Fudan University, Shanghai, China; 4 Department of Epidemiology, School of Public Health, Fudan University, Shanghai, China; Affiliated Hospital of Nantong University, CHINA

## Abstract

To analyze the post-COVID-19 construction and management of fever clinics targeted to prevention and control of healthcare-associated respiratory viral infections in medical institutions at all levels in China, and to provide a basis for promoting their standardized construction, we conducted this survey on the construction of fever clinics in 429 medical institutions of Jiangsu Province from July to December 2020. Contents of the questionnaire included the general situation of medical institutions, the construction status and future construction plans of fever clinics. We find the construction rate of fever clinic in medical institutions of Jiangsu province was 75.3%. All construction indicators, quality management systems and processes fail to fully meet the requirements of documents and standards. Jiangsu province actively promotes the construction of fever clinic layout, but there is still a gap with the construction standard. As a result, it is necessary to further promote standardized construction of fever clinic, and necessary financial input should be increased to expand all constructions of fever clinic in primary medical institutions.

## Introduction

The coronavirus disease 2019 (COVID-19) pandemic has caused a sharp increase in hospitalizations for pneumonia, which is caused by the novel severe acute respiratory syndrome coronavirus 2 (SARS-CoV-2). Patients infected with SARS-CoV-2 may have various symptoms like cough and fever, or even be asymptomatic [1–3]. Fever clinics (FCs) are specifically geared toward treating people who have a fever, defined as a temperature of 100 degrees Fahrenheit or higher, or other COVID-19 symptoms, such as extreme fatigue or severe cough [4]. People with these symptoms also can get tested for COVID-19 at a fever clinic. Setting up FCs is considered as one of the most effective public health interventions to control the spread of the epidemic, but this work requires experience and lacks scientific and effective guidance domestically [5].

Since the original implementation of the FC system by the National Health Commission of the People’s Republic of China during the SARS epidemic in 2003, FCs have been providing prompt assessment, management, laboratory examination and decision-making for the potential infected cases especially at the early stage of an epidemic, especially during COVID-19 combat in China [6]. Guided by the principle of “early assessment, early detection, and early isolation”, fever clinic is the fundamental resource and critical sentry for triaging suspected cases and minimize the risk of cross infection in the hospital [7]. National and provincial health administrative departments have successively issued relevant management requirements and construction standards for FCs, and proposed requirements for building layout, working process, standardized management and personal protection of FCs inland [8]. Large amounts of investigations of FCs in several cities in China implies that, with rapid screening system and upgraded protocol, FCs have effectively helped to quickly identify and isolate patients with COVID-19 and have prevented this disease from spreading nationwide, though the workload of emergency department has significantly increased after the pandemic outbreak [4, 5, 9–11]. However, the number of patients greatly exceeds the number of physicians, which discloses the problems such as the inadequate and unbalanced supply of medical services [9]. Thousands of healthcare providers in medical institutions even have been infected [10]. Therefore, fever clinics have failed to function normally as expected along with the havoc of pandemic.

Jiangsu Province has initiated wartime control measures for the epidemic [12]. It has been reported that although pertinent specifications and regulations for FCs have been issued, some indexes such as architectural composition have failed to meet the standard requirements [13]. The current limitations of FCs in Jiangsu Province are rarely studied, and more evidence of standardizing the construction of FCs and strengthening the scientific prevention and control mechanism of COVID-19 is needed. We conducted a special survey on the current situation of fever clinic construction at all levels in Jiangsu Province by using a unified questionnaire. Numerous indexes were included in this survey, such as setting up fever clinic or not, architectural composition, regional setting and future construction planning and so on. We hope to explore the post-COVID-19 construction and management of fever clinics targeted to prevention and control of healthcare-associated respiratory viral infections in medical institutions at all levels, and to provide the basis for expanding the coverage and standardized construction of fever clinic.

## Methods and materials

### Research object

Under the organization of the Jiangsu Hospital Infection Management Committee (JSHIMC), from July to December 2020, the survey was conducted on the construction of fever clinics and infectious diseases departments in 429 medical institutions at all levels in Jiangsu Province, including level I, level II and level III, public, private and military hospitals, comprehensive and specialized hospitals, and community health service centers (stations) / outpatient departments.

### Research methods

According to the standards for the construction of fever clinics and infectious diseases departments, “Construction standard for fever clinics and infectious diseases departments of medical institutions in Jiangsu Province (2020 Edition)” (hereinafter referred to as the “acceptance standards”) issued by the health commission of Jiangsu Province, the construction of fever clinics in 429 medical institutions in the province was investigated and evaluated. The specific contents include general situation of medical institutions, current situation and future construction plan of fever clinic. Two full-time doctors, engaged in nosocomial infection management, worked as investigators and quality analysis controllers to ensure the authenticity and reliability of data acquisition.

### Statistical analysis

Excel 2007 was used to establish the database and import data. Categorical variables were described by rate and constituent ratio. The Chi-square test (or Fisher exact test where necessary) was used to compare proportions between different groups. All tests were 2-tailed and P values <0.05 were considered as statistically significant. Statistical analyses were conducted using SPSS 20.0 software package (IBM Company, New York).

## Results

### Characteristics of medical institutions

Characteristics of medical institutions are shown in Table 1. A total of 429 medical institutions were involved in this study. All the hospitals (113 tertiary hospitals, 232 secondary hospitals, and 84 primary hospitals) were located in 13 cities of Jiangsu Province, China. More than half of hospitals were general hospitals (66.0%) and public hospitals (72.7%). In addition, more than three-fourth of hospitals had fever clinics (75.3%). However, only 33.3% hospitals had departments of infectious diseases. Moreover, the total number of beds were mostly less than 1000 among these medical institutions.

**Table 1:** Characteristics of medical institutions of Jiangsu Province (n = 429).

Baseline		n	%
Level	Tertiary	113	26.3
	Secondary	232	54.1
	Primary	84	19.6
Type	General	283	66.0
	Specialized	98	22.8
	Others	48	11.2
Nature	Public	312	72.7
	Private	116	27.0
	Military	1	0.2
Department of infectious diseases	Yes	143	33.3
	No	286	66.7
Fever clinic	Yes	323	75.3
	No	106	24.7
Number of beds	>3000	5	1.2
	2000<n≤3000	2	0.5
	1000<n≤2000	26	6.1
	500<n≤1000	81	18.9
	100<n≤500	193	45.0
	≤100	122	28.4

### Characteristics of fever clinics

#### General characteristics

General characteristics of fever clinics are detailed in Table 2. In this survey, the majority of fever clinics were established since 2003, especially for primary hospitals. Of the 10.9% tertiary hospitals and 41.9% secondary hospitals began to build fever clinics since 2020. Of note that the location of fever clinics was mostly set independently, outside medical buildings. The building area of fever clinics was usually between 100 m^2^ and 500 m^2^ in tertiary hospital (55.5%), secondary hospital (75.3%) and primary hospital (48.1%). In terms of pediatric fever clinic, it was usually set as a fixed consultation room of pediatric clinics in tertiary hospital (46.4%) and secondary hospital (35.5%), while in some primary hospitals (48.1%), internal medicine department served as fever clinics as well.

**Table 2:** General characteristics of fever clinics (n = 323).

Items		Level of medical institutions						χ^2^	*P Value*
		Tertiary n (%)		Secondar n (%)		Primary n (%)			
Building time	Before 2003	31	(28.2)	19	(10.2)	0	(0.0)	45.526	<0.001
	2003 to 2019	67	(60.9)	89	(47.8)	13	(48.1)		
	2020	12	(10.9)	78	(41.9)	14	(51.9)		
Location	in medical building	22	(20.0)	39	(21.0)	3	(11.1)	21.491	<0.001
	In infectious diseases building	13	(11.8)	1	(0.5)	1	(3.7)		
	in independent building	75	(68.2)	146	(78.5)	23	(85.2)		
Building area (m^2^)	≤100	4	(3.6)	25	(13.4)	13	(48.1)	72.937	<0.001
	100–500	61	(55.5)	140	(75.3)	13	(48.1)		
	500–1000	28	(25.5)	15	(8.1)	1	(3.7)		
	1000–1500	13	(11.8)	4	(2.2)	0	(0.0)		
	>1500	4	(3.6)	2	(1.1)	0	(0.0)		
Pediatric fever clinic	In pediatric clinic, no fixed rooms	11	(10.0)	45	(24.2)	1	(3.7)	70.457	<0.001
	In pediatric clinic, fixed rooms	51	(46.4)	66	(35.5)	2	(7.4)		
	In emergency area, no fixed rooms	3	(2.7)	2	(1.1)	0	(0.0)		
	In emergency area, fixed rooms	14	(12.7)	14	(7.5)	1	(3.7)		
	In clinic of Internal medicine	5	(4.5)	22	(11.8)	13	(48.1)		
	In fever clinic area, fixed room	19	(17.3)	13	(7.0)	6	(22.2)		
	No pediatric fever clinic	7	(6.4)	24	(12.9)	4	(14.8)		

#### Layout of fever clinics

Description of layout of fever clinics is presented in Table 3. In this study, almost all fever clinics set up three zones and two passageways in 109 (99.1%) tertiary hospitals, 182 (97.8%) secondary hospitals and 22 (81.5%) primary hospitals. In addition, the majority of fever clinics had duty room, bathroom, buffer room, patient-only corridor, patients’ waiting area and sampling room. However, there were only 30.9% tertiary hospitals and 19.9% secondary hospitals equipped CT rooms in the fever clinics. The number of consulting rooms and observing rooms was usually 1 to 3 respectively. Moreover, the proportion of non-touch water taps in fever clinics of tertiary and secondary hospitals were 91.8% and 87.1% respectively, while that in primary hospitals was only 55.6%.

**Table 3:** Characteristics of layout of fever clinics (n = 323).

Items		Level of medical institutions						χ^2^	*P Value*
		Tertiary n (%)		Secondary n (%)		Primary n (%)			
Three zones and two passageways	Yes	109	(99.1)	182	(97.8)	22	(81.5)	23.714	<0.001
	No	1	(0.9)	4	(2.2)	5	(18.5)		
Patient-only corridor	Yes	64	(58.2)	112	(60.2)	14	(51.9)	24.019	<0.001
	No	45	(40.9)	70	(37.6)	8	(29.6)		
Bathroom	No	13	(11.8)	27	(14.5)	13	(48.1)	22.006	<0.001
	Yes	97	(88.2)	159	(85.5)	14	(51.9)		
Duty room	No	15	(13.6)	53	(28.5)	10	(37.0)	11.003	0.004
	Yes	95	(86.4)	133	(71.5)	17	(63.0)		
Buffer room	No	6	(5.5)	15	(8.1)	12	(44.4)	38.142	<0.001
	Yes	104	(94.5)	171	(91.9)	15	(55.6)		
Patients’ waiting area	No	3	(2.7)	20	(10.8)	8	(29.6)	18.758	<0.001
	Yes	107	(97.3)	166	(89.2)	19	(70.4)		
Sampling room	No	16	(14.5)	35	(18.8)	10	(37.0)	7.160	0.028
	Yes	94	(85.5)	151	(81.2)	17	(63.0)		
CT room	No	76	(69.1)	149	(80.1)	26	(96.3)	10.719	0.005
	Yes	34	(30.9)	37	(19.9)	1	(3.7)		
Number of consulting rooms	1–3	101	(91.8)	181	(97.3)	25	(92.6)	6.874	0.143
	4–10	9	(8.2)	4	(2.2)	2	(7.4)		
	>10	0	(0.0)	1	(0.5)	0	(0.0)		
Number of observing rooms	0	1	(0.9)	2	(1.1)	2	(7.4)	44.035	<0.001
	1–3	75	(68.2)	171	(91.9)	23	(85.2)		
	4–10	20	(18.2)	12	(6.5)	2	(7.4)		
	>10	14	(12.7)	1	(0.5)	0	(0.0)		
Water taps	Some were non-touch	2	(1.8)	10	(5.4)	6	(22.2)	26.478	<0.001
	All were non-touch	101	(91.8)	162	(87.1)	15	(55.6)		
	All were touch	7	(6.4)	14	(7.5)	6	(22.2)		

#### Infection control and quality control

Description of infection control and quality control of fever clinics are presented in Table 4. In the current investigation, among tertiary and secondary hospitals, many fever clinics affiliated to medical offices (45.5% vs. 47.8%) or outpatient department (32.7% vs.17.2%). Different in primary hospitals that there were 48.1% medical offices and 29.6% departments of nosocomial infection managed fever clinics. Window opening was the main method to manage air distribution in fever clinics among medical institutions (47.3% tertiary hospitals vs. 62.4% secondary hospitals vs. 88.9% primary hospitals). The majority of fever clinics had check list of infection control (89.1% tertiary hospitals vs. 80.6% secondary hospitals vs. 66.7% primary hospitals), self-examination (90.0% tertiary hospitals vs. 70.4% secondary hospitals vs. 70.4% primary hospitals), multi-sectoral collaboration and continuous quality improvement (92.7% tertiary hospitals vs. 82.3% secondary hospitals vs. 77.8% primary hospitals).

**Table 4:** Description of infection control and quality control of fever clinics(n = 323).

Items		Level of medical institutions						χ^2^	*P Value*
		Tertiary n (%)		Secondary n (%)		Primary n (%)			
Department in charge	Medical department	50	(45.5)	89	(47.8)	13	(48.1)	27.689	0.002
	Nursing department	3	(2.7)	15	(8.1)	4	(14.8)		
	Outpatient department	36	(32.7)	32	(17.2)	1	(3.7)		
	Department of nosocomial infection	9	(8.2)	30	(16.1)	8	(29.6)		
	Others	6	(5.5)	7	(3.8)	0	(0.0)		
	Not clear	6	(5.5)	13	(7.0)	1	(3.7)		
Air distribution	Mechanical ventilation in three zones, fresh air from clean zone to contaminated zone	36	(32.7)	28	(15.1)	2	(7.4)	24.135	<0.001
	Mechanical ventilation only in contaminated zone, no fresh air	22	(20.0)	42	(22.6)	1	(3.7)		
	Only window opening	52	(47.3)	116	(62.4)	24	(88.9)		
Air conditioning system maintenance and filter replacement	Normal	92	(83.6)	139	(74.7)	18	(66.7)	10.830	0.029
	Frequency is unnormal	18	(16.4)	44	(23.7)	7	(25.9)		
	Never	0	(0.0)	3	(1.6)	2	(7.4)		
Check list of infection control	Yes	98	(89.1)	150	(80.6)	18	(66.7)	8.381	0.015
	No	12	(10.9)	36	(19.4)	9	(33.3)		
Self-examination	Yes	99	(90.0)	131	(70.4)	19	(70.4)	15.742	<0.001
	No	11	(10.0)	55	(29.6)	8	(29.6)		
Multisectoral collaboration and continuous quality improvement	Yes	102	(92.7)	153	(82.3)	21	(77.8)	7.488	0.024
	No	8	(7.3)	33	(17.7)	6	(22.2)		

## Discussion

Since the outbreak of COVID-19, the Ministry of Health of the People’s Republic of China has released several documents to specify the establishment of fever clinics [14–16]. The current study showed that most medical institutions in Jiangsu Provence have established relatively standardized fever clinics, laying a solid foundation for the regular prevention and control of COVID-19. However, some common problems are also found, which need to be further perfected and improved.

Compared with previous studies [17], this study combining the latest file, included in more widely and diverse medical institutions, such as community health service centers, out-patient departments and local hospitals, which makes up for the shortcomings of the previous survey and provides a further basis for perfecting the emergency management system of public health emergencies in China. Briefly, according to our study, more than 90 percent of secondary hospitals and 80 percent of primary hospitals didn’t have the required number of observation rooms in fever clinics, which should be improved in order to respond to the sporadic local clusters of COVID-19 outbreaks [18]. More than half of hospitals haven’t established CT departments, especially for fever outpatient patients. There is still a large risk of nosocomial infection induced by shared CT machines [19–21]. In addition, more than 90 percent of fever clinics of medical institutions were not equipped with air disinfection apparatus in the air conditioning ventilation system and didn’t maintain air conditioning systems regularly, which has laid a huge hidden danger for nosocomial infection of airborne infectious diseases. What’s more, the proportion of fever clinics in primary medical institutions that are not fully equipped with hands-free faucets is as high as 44.4%. All these results are still far from the qualified standard required by the document.

At present, most foreign countries are adopting the strategy of opening up to the epidemic, while China is adopting the dynamic zero-out strategy. To further improve our epidemic prevention and control policies, health authorities should incorporate the construction of fever clinics into the prevention and control system of public health emergencies and conduct on-site supervision and assessment of medical institutions at all levels from time to time. Standardized and information-based measures should be taken to establish an integrated management and facilities for screening, diagnosis, treatment and prevention and control of infectious diseases [17]. In the case of local clusters of epidemic diseases, fever clinics should immediately execute independently upgraded management, under the direct responsibility of the vice president in charge of medical work. More importantly, the government should increase the financial input for the standardized renovation and expansion of fever clinics. It also should be included in the category of public health professional service institutions, and the necessary funds should be guaranteed by all levels of finance [22, 23].

However, there are still several limitations of this study. Firstly, we only surveyed hospitals at all levels in Jiangsu Province. Jiangsu is a relatively developed province in economy and medicine, which could not reflect the situation of our whole country. Secondly, this study mostly focused on the hardware facilities of medical institution but lack data of the situation of hospital management. Next, we will further perform a more comprehensive survey including both hardware facilities and hospital management in our whole country but not individual province.

## Conclusion

To conclude, this report summarized the first data collected on the status of fever clinics in Jiangsu Province, which demonstrated that most medical institutions in Jiangsu Province have established relatively standardized fever clinics while some common problems are also existing. In the future, we need to conduct a more comprehensive survey of fever clinics nationwide. Only when we continuously make up for the insufficiency of fever clinic construction exposed during the epidemic could we better prevent and control healthcare-associated respiratory viral infections in the post- COVID-19 era.
